# Sp1 is Involved in Vertebrate LC-PUFA Biosynthesis by Upregulating the Expression of Liver Desaturase and Elongase Genes

**DOI:** 10.3390/ijms20205066

**Published:** 2019-10-12

**Authors:** Yuanyou Li, Jianhong Zhao, Yewei Dong, Ziyan Yin, Yang Li, Yang Liu, Cuihong You, Óscar Monroig, Douglas R. Tocher, Shuqi Wang

**Affiliations:** 1 Joint Laboratory of Guangdong Province and Hong Kong Region on MBCE, College of Marine Sciences, South China Agricultural University, Guangzhou 510642, China; sunshinewonder@163.com; 2 Guangdong Provincial Key Laboratory of Marine Biotechnology, Shantou University, Shantou 515063, China; 15jhzhao@alumni.stu.edu.cn (J.Z.); 15zyyin@alumni.stu.edu.cn (Z.Y.); liyang091616@163.com (Y.L.); 15yliu7@alumni.stu.edu.cn (Y.L.); chyou@stu.edu.cn (C.Y.); 3 Instituto de Acuicultura Torre de la Sal (IATS-CSIC), Ribera de Cabanes, 12595 Castellón, Spain; oscar.monroig@csic.es; 4 Institute of Aquaculture, Faculty of Natural Sciences, University of Stirling, Stirling FK9 4LA, UK; d.r.tocher@stir.ac.uk

**Keywords:** Sp1, Δ6/Δ5 *fads2*, Δ4 *fads2*, *elovl5*, LC-PUFA biosynthesis, rabbitfish *Siganus canaliculatus*

## Abstract

The rabbitfish *Siganus canaliculatus* was the first marine teleost demonstrated to have the ability for the biosynthesis of long-chain (≥C20) polyunsaturated fatty acids (LC-PUFA) from C18 PUFA precursors, and all the catalytic enzymes including two fatty acyl desaturase 2 (Δ4 Fads2 and Δ6/Δ5 Fads2) and two elongases (Elovl4 and Elovl5) have been identified, providing a good model for studying the regulatory mechanisms of LC-PUFA biosynthesis in fish. Stimulatory protein 1 (Sp1) has been speculated to be a vital transcription factor in determining the promoter activity of Fads-like genes in fish, however its regulatory effects on gene expression and LC-PUFA biosynthesis have not been demonstrated. Bioinformatic analysis predicted potential Sp1 binding sites in the promoters of the rabbitfish Δ6/Δ5 *fads2* and *elovl5*, but not in Δ4 *fads2* promoter. Here we cloned full-length cDNA of the rabbitfish *sp1* gene, which encoded a putative protein of 701 amino acids, and was expressed in all tissues studied with highest levels in gill and eyes. The dual luciferase reporter assay in HepG2 line cells demonstrated the importance of the Sp1 binding site for the promoter activities of both Δ6/Δ5 *fads2* and *elovl5*. Moreover, the electrophoretic mobility shift assay confirmed the direct interaction of Sp1 with the two promoters. Insertion of the Sp1 binding site of Δ6/Δ5 *fads2* promoter into the corresponding region of the Δ4 *fads2* promoter significantly increased activity of the latter. In the *Siganus canaliculatus* hepatocyte line (SCHL) cells, mRNA levels of Δ6/Δ5 *fads2* and *elovl5* were positively correlated with the expression of *sp1* when *sp1* was overexpressed or knocked-down by RNAi or antagonist (mithramycin) treatment. Moreover, overexpression of *sp1* also led to a higher conversion of 18:2n−6 to 18:3n−6, 18:2n−6 to 20:2n−6, and 18:3n−3 to 20:3n−3, which related to the functions of Δ6/Δ5 Fads2 and Elovl5, respectively. These results indicated that Sp1 is involved in the transcriptional regulation of LC-PUFA biosynthesis by directly targeting Δ6/Δ5 *fads2* and *elovl5* in rabbitfish, which is the first report of Sp1 involvement in the regulation of LC-PUFA biosynthesis in vertebrates.

## 1. Introduction

Long-chain (≥C_20_) polyunsaturated fatty acids (LC-PUFA), such as eicosapentaenoic (EPA; 20:5n−3), arachidonic (ARA, 20:4n−6), and docosahexaenoic (DHA; 22:6n−3) acids play important roles in growth, development, and reproduction in vertebrates, being specifically involved in maintenance of cellular membrane structure, energy metabolism, gene regulation and cellular signaling, and promoting cardiovascular health and immune function [1,2]. Fish, especially marine species, are major sources of LC-PUFA in the human diet [3]. However, with overfishing and the degradation of the marine environment, natural wild fishery stocks have reduced sharply. The declining capture fisheries has turned attention to farmed marine fish as the major source of LC-PUFA. Thus, much attention has been focused on elucidating the regulatory mechanisms of LC-PUFA biosynthesis, in order to maximize endogenous production in marine fish.

While LC-PUFA are important for normal growth and development of all fish, the biosynthetic capacity differs between species [4]. All the teleost fatty acyl desaturases (*fads2)* genes cloned to date are homologous to mammalian *fads2*, but their substrate specificities differ among species and monofunctional and bifunctional desaturases with Δ4, Δ5, and Δ6 activities have been described [5]. Moreover, elongases of very long-chain fatty acids *(elovl*) encoding genes with relevant roles in the biosynthesis of LC-PUFA in teleosts include *elovl2*, *elovl4*, and *elovl5*, of which *elovl4* and *elovl5* are present in virtually all teleosts [5]. In general, freshwater fish and salmonid species can convert the C_18_ PUFA precursors, α-linolenic acid (18:3n−3; ALA) and linoleic acid (18:2n−6; LA), to LC-PUFA through a series of desaturation and elongation reactions catalyzed by Fads2 and Elovl, whereas most marine teleosts lack or have very limited capability [6,7,8]. Consequently, essential fatty acid (EFA) requirements of freshwater fish can be satisfied by ALA and LA, while marine fish require dietary LC-PUFA. Accordingly, in aquaculture production, vegetable oils rich in ALA and LA can be used as dietary lipid sources for freshwater fish, while fish oil rich in LC-PUFA is required in feed for marine fish to meet EFA requirements for normal growth. The limited supplies of fish oil resources and their high price restricts the sustainable development of the mariculture industry. Therefore, it is necessary and important to understand the regulatory mechanisms of LC-PUFA biosynthesis in fish so as to develop methods to optimize the endogenous production (biosynthesis) of LC-PUFA with the aim to reduce the reliance of the aquaculture industry on fish oil.

It is understood that the capability of fish for LC-PUFA biosynthesis depends largely on the expression and/or activities of key enzymes involved in the biosynthetic pathway [5,9,10]. At a transcriptional level, sterol regulatory element binding proteins 1 (Srebp-1) and peroxisome proliferator-activated receptors (Ppars) are major transcription factors (TF) of genes for key enzyme involved in lipid metabolism including LC-PUFA biosynthesis [11]. While two forms of Srebp-1, i.e., Srebp-1a and -1c, have been characterized in mammals [12], only a single form of Srebp-1 was characterized in fish, and this demonstrated to be involving two subtypes of Pparα (namely Pparα1 and Pparα2) in some fishes [13,14,15], four Pparβ subtypes in Atlantic salmon (*Salmo salar*) [16], and three Ppar subtypes in rabbitfish (*Siganus canaliculatus*) (Pparα, Pparβ, and Pparγ) [17]. It was reported that Pparα up-regulated *fads2* promoter activity in rainbow trout (*Oncorhynchus mykiss*) and Japanese seabass (*Lateolabrax japonicus*) [18], and Pparγ is involved in the transcriptional regulation of Δ6/Δ5 *fads2* in the liver of *S. canaliculatus* [19].

In the recent years, stimulatory protein 1 (Sp1) binding sites were found in the gene promoter of human Δ6 *fads2* [6], pig *elovl6* [20], and bovine *elovl7* [21]. In fish, the Δ6 *fads2* promoter of Atlantic salmon showed stronger promoter activity than that of Atlantic cod (*Gadus morhua*) associated with the presence of the Sp1 binding site in the former [22]. Furthermore, the lack of Sp1 binding sites in the promoters of the *fads2* gene of *L. japonicus*, *Dicentrarchus labrax,* and *Epinephelus coioides* was associated with lower activity of the promoters [23]. These results suggested that Sp1 could be involved in the regulation of LC-PUFA biosynthesis in teleost fish by activating promoter activities of genes encoding key enzymes. However, no direct evidence has been presented, and the underlying functions of Sp-1 and the mechanisms involved are not clear.

Rabbitfish *S. canaliculatus* is a commercially important marine teleost fish widespread along the Indo-West Pacific coast and also known as one of the mainly harvested fish species. It is naturally herbivorous, consuming algae and seagrass; however, they can also feed on compound feed or trash fishes after brief domestication with them. It is noteworthy that rabbitfish was the first marine teleost demonstrated to have capability for LC-PUFA biosynthesis from C_18_ precursors [24]. Genes encoding key enzymes for LC-PUFA biosynthesis including Δ4 Fads2, bifunctional Δ6/Δ5 Fads2, Elovl4, and Elovl5 were functionally characterized in this species, which provides a good model for studying the regulatory mechanisms of LC-PUFA biosynthesis in teleosts [24,25]. In addition, bioinformatic analysis predicted Sp1 binding sites in the promoters of rabbitfish Δ6/Δ5 *fads2* and *elovl5*, but Sp1 binding sites were absent in the promoters of Δ4 *fads* and *elovl4*. Furthermore, in a recent study, inserting the Sp1 binding site of rabbitfish Δ6/Δ5 *fads*2 promoter into the corresponding region of *E. coioides fads*2 promoter demonstrated the importance of the Sp1 binding site in determining *fads2* promoter activity [23]. However, until now, no study has directly demonstrated the role of Sp1 in the regulation of LC-PUFA biosynthesis in any vertebrate including fish, and thus the present study aimed to clarify this in rabbitfish. Therefore, the *sp1* gene was cloned, and its function in the regulation of LC-PUFA biosynthesis was investigated by determining the effects of Sp1 on the expression of Δ6/Δ5 *fads*2 and *elovl5* genes, and on the conversion of C_18_ fatty acid precursors to LC-PUFA. The data obtained increased our understanding of the regulatory mechanisms of LC-PUFA biosynthesis in vertebrates and will contribute to the optimization and/or enhancement of LC-PUFA biosynthesis in teleosts.

## 2. Results

### 2.1. Cloning and Characterization of Sp1

**Figure 1:**
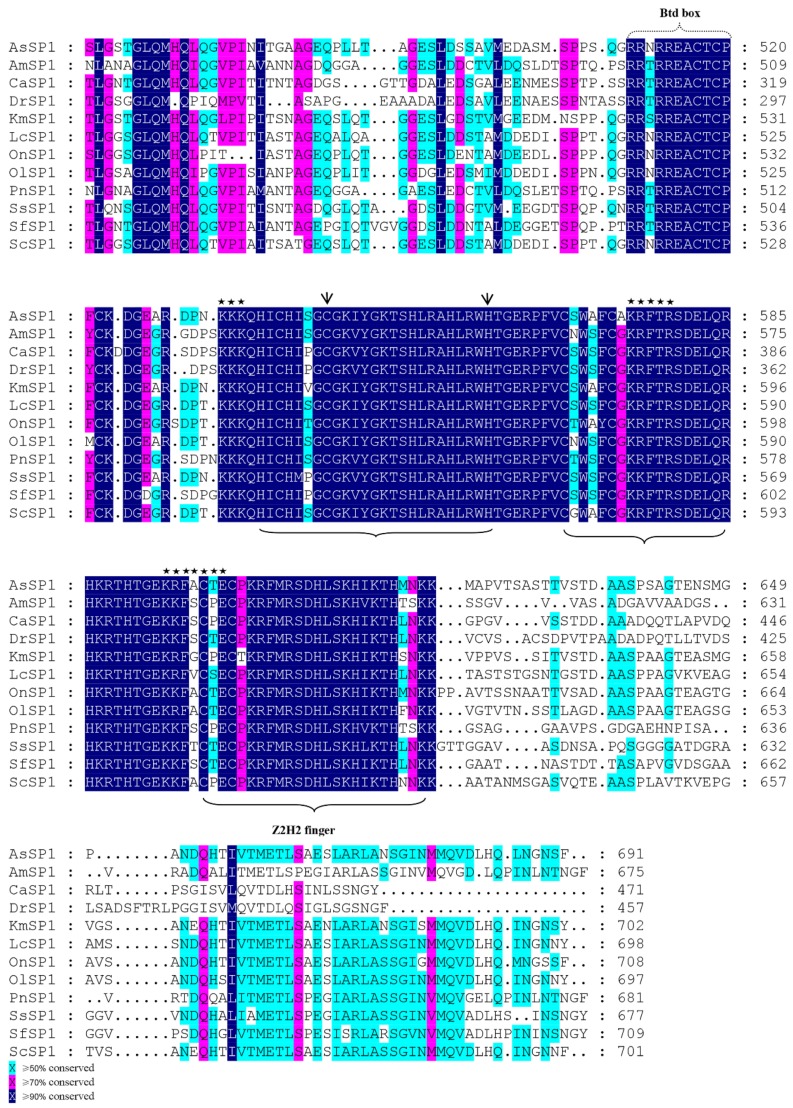
Alignments of the deduced amino acid (aa) sequences of Sp1 Btd box and zinc finger domains from *Siganus canaliculatus* (ScSP1) and other fish species (*Larimichthys crocea*, LcSP1, XP_010730401.1; *Salmo salar*, SsSP1, XP_013989519.1; *Aphyosemion striatum* AsSP1, SBP16265.1; *Astyanax mexicanus*, AmSP1, XP_007248419.1; *Kryptolebias marmoratus*, KmSP1, XP_017264015.1; *Oreochromis niloticus*, OnSP1, XP_019214905.1; *Oryzias latipes*, OlSP1, XP_004068725.1; *Pygocentrus nattereri*, PnSP1, XP_017548304.1; *Scleropages formosus*, SfSP1, XP_018597836.1; *Danio rerio*, DrSP1, AAH67713.1). The black and gray boxes indicate identical and similar aa residues, respectively. The dotted-line brackets indicate Btd box. The solid braces denote zinc finger domains. The pentagrams and arrows indicate potential phosphorylation sites and Zn binding sites.

**Figure 2:**
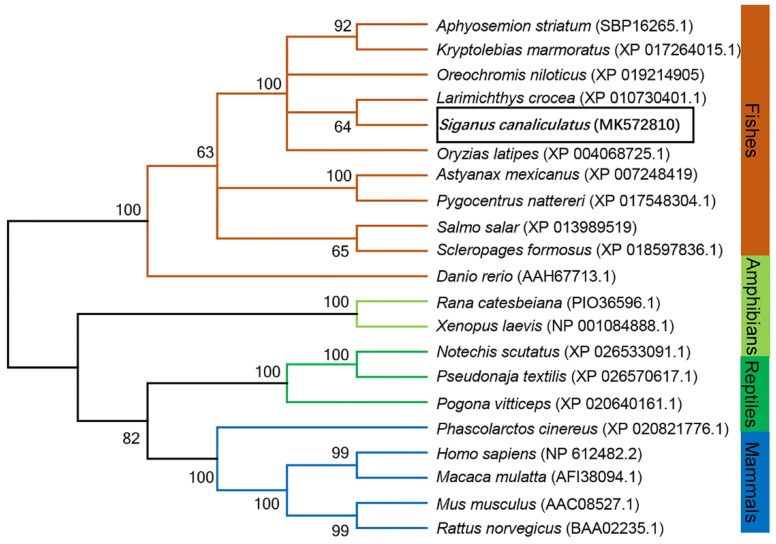
Phylogenetic analysis of the deduced amino acid sequences of Sp1 proteins from rabbitfish and other species with the neighbor-joining method by using MEGA 5.0 Version. Bootstrap values were obtained from 1000 repetitions and illustrated as percentages at the nodes. The sequences of rabbitfish Sp1 are boxed.

**Figure 3:**
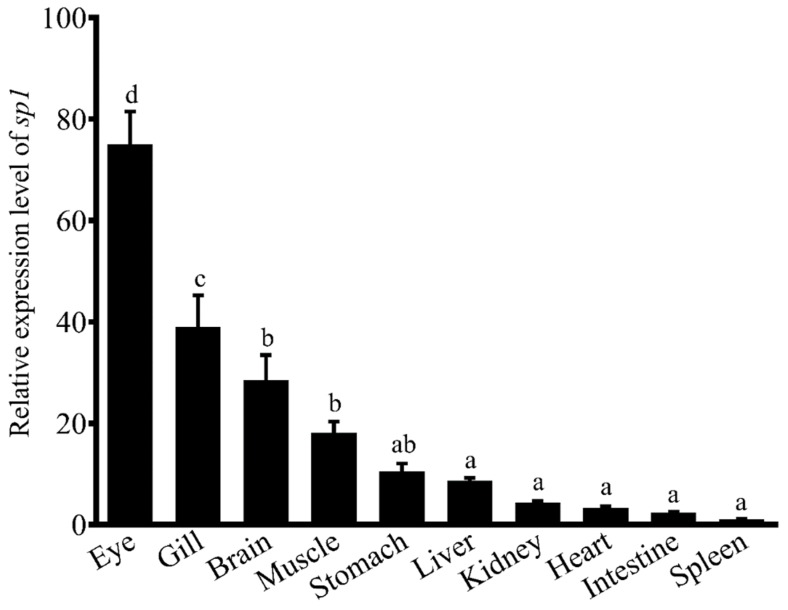
Tissue-specific expression of rabbitfish *sp1* by quantitative PCR. The mRNA levels of rabbitfish *sp1* in each tissue are separately presented as the fold change from the level in the intestine by using the comparative threshold cycle method. Relative expression of *sp1* were quantified for each transcript and were normalized with 18S by 2^−ΔΔCt^ method. Results are means ± SEM (*n* = 6), bars without sharing a common letter indicate significant differences (*p* < 0.05) among tissues as determined by one-way ANOVA followed by Tukey’s multiple comparison test.

A 3724 bp length of rabbitfish *sp1* cDNA was cloned (GenBank accession no. MK572810), which contained a 2106 bp open-reading frame (ORF) that encoded a protein of 701 amino acids, which contains all the typical structural characteristics of Sp1 including one Sp box at the N-terminus (Figure S1), Btd box and three zinc finger domains (Figure 1) at the C-terminus and potential phosphorylation sites throughout the sequence. Phylogenetic analysis of Sp1 among vertebrates showed that the rabbitfish Sp1 was most closely clustered to that of large yellow croaker (*Larimichthys crocea*), and more distantly from freshwater fish and mammals (Figure 2). In addition, rabbitfish *sp1* was expressed in all 10 tissues tested and was particularly abundant in eyes and gills, which showed significantly higher expression levels than those in other tissues (Figure 3).

**Figure 4:**
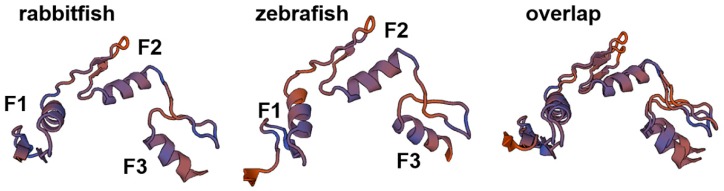
The predicted three-dimensional structures of the Sp1 protein DNA-binding zinc finger domains in the rabbitfish (*S. canaliculatus*) and zebrafish (*D. rerio*), and their overlap. The predicted domain structures were modeled using the on-line program SWISS-MODEL Automated Protein Modeling Mode (http://swissmodel.expasy.org/).

The three-dimensional structure analysis of Sp1 protein between rabbitfish (*S. canaliculatus*) and zebrafish (*Danio rerio*) showed that both of contained structurally comparable DNA-binding zinc finger domains. Zebrafish (residues 447–533) and rabbitfish Sp1 peptide (residues 543–626) contain three α-helixes (F1, F2, and F3) and anti-parallel β-sheets zinc finger domains with highly similar spatial arrangement. The similarity between the two domains was more evident when the structures were overlapped (Figure 4).

### 2.2. The Sp1 Element in the Core Promoter Region of Δ6/Δ5 fads2 and elovl5 is Essential for Promoter Activity

**Figure 5:**
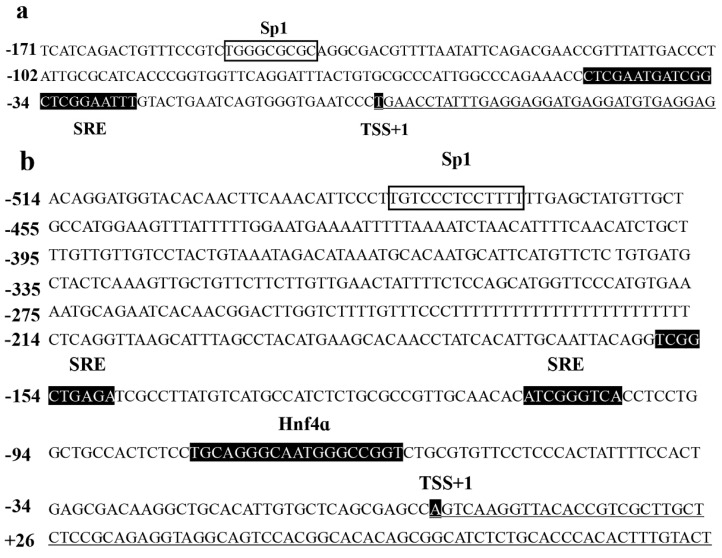
The nucleotide sequence and predicted binding sites for Sp1 in the core region of rabbitfish Δ6/Δ5 *fads2* (**a**) and *elovl5* (**b**) promoters. Numbers are given relative to the first base of the transcription start site (TSS, +1). Potential transcription binding motifs are marked in black or open boxes for Sp1. The bases underlined are downstream sequence of TSS.

**Figure 6:**
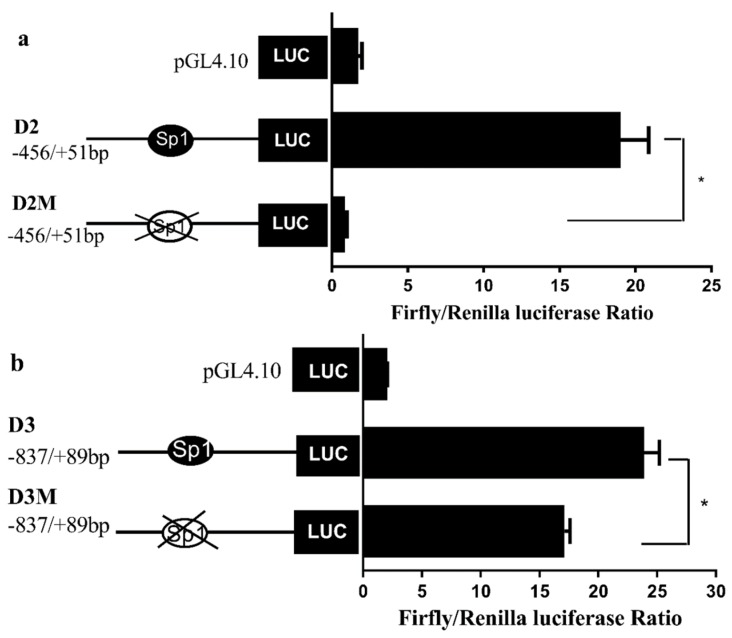
Effects of Sp1 site-directed mutations on the promoter activity of *S. canaliculatus* Δ6/Δ5 *fads*2 (**a**) and *elovl5* (**b**) detected in HepG2 cells. The negative control pGL4.10 is an empty vector with no promoter sequence upstream the reporter gene. The y-axis is the Firefly/Renilla luciferase ratio, while the x-axis stands for different reporter vector. Data were mean ± SEM from six independent experiments and asterisks represent significant differences (*p* < 0.05).

Both the core promoter regions of the Δ6/Δ5 *fads2* and *elovl5* contained GC rich sites. Using the bioinformatics software TRANSFAC^®^ and TF binding^®^, Sp1 binding sites were predicted in the promoter regions of rabbitfish Δ6/Δ5 *fads2* (−159 to −137 bp) and *elovl5* (−491 to −468 bp), respectively (Figure 5a, b). To evaluate the role of the Sp1 binding elements in determining the promoter activity of Δ6/Δ5 *fads2* and *elovl5*, targeted mutations within these sites were carried out as shown in Table 1. The results showed that promoter activities of both Δ6/Δ5 *fads2* (Figure 6a) and *elovl5* (Figure 6b) were significantly decreased after Sp1 binding elements were mutated, which indicated that the Sp1 binding site at position −159 to −137 bp of Δ6/Δ5 *fads2* and −491 to −468 bp of *elovl5* could be important for core promoter activity.

**Figure 7:**
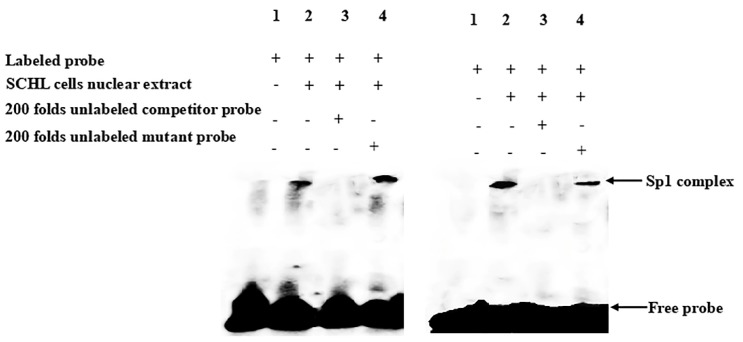
The electrophoretic mobility shift assay (EMSA) of Δ6/Δ5 *fads2* (left) and *elovl5* (right) probes with *S. canaliculatus* hepatocytes nuclear proteins. Each lane is represented as lane 1, negative control; lane 2, nucleus proteins reactions; lane 3, unlabeled probe competing reactions; lane 4, unlabeled mutant probe competing reactions. “+” means that the corresponding material in the row has been added, and “−” means that the material is not added.

To determine whether Sp1 binds to the Δ6/Δ5 *fads2* and *elovl5* promoter regions via specific Sp1 binding sites, electrophoretic mobility shift assay (EMSA) was performed. The results showed that the DNA–protein complex was detected when nuclear protein extracts of *Siganus canaliculatus* hepatocyte line (SCHL) cells were incubated with the double-stranded oligonucleotide probe containing proximal Sp1-binding site (Figure 7). Unlabeled competitor probe could compete with the binding reaction (lane 3) and un-labeled mutant competitor probe could not compete in the reaction (lane 4). Taken together, the data demonstrated that Sp1 could directly bind to the promoters of both Δ6/Δ5 *fads2* and *elovl5*, and thus potentially regulate Δ6/Δ5 *fads2* and *elovl5* transcription.

### 2.3. Effect of Sp1 Binding Site Insertion on Δ4 fads2 Promoter Activity

**Figure 8:**
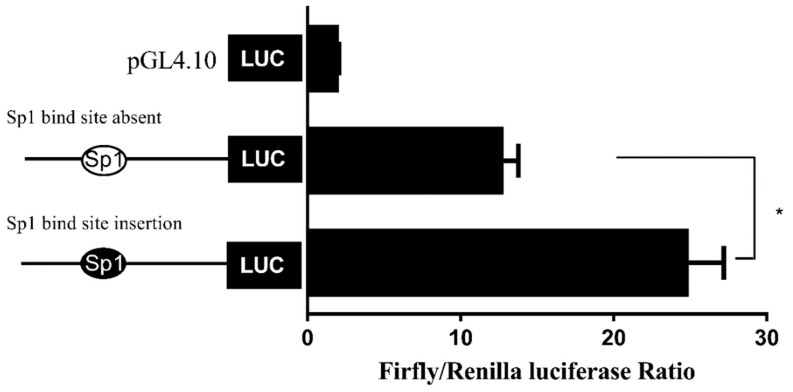
Effect of Sp1 binding site insertion on the activity of Δ4 *fads2* promoter. The Sp1 binding site of Δ6/Δ5 *fads2*, which was inserted into the corresponding area of Δ4 *fads2* is indicated with a black ellipse. Results are means ± SEM (*n* = 6), * means significant difference (*p* < 0.05).

The Sp1 binding site was predicated in the promoter of Δ6/Δ5 *fads2*, but absent in that of Δ4 *fads2* of *S*. *canaliculatus* [23]. To test whether the absence of Sp1 binding site was related to the lower promoter activity of Δ4 *fads2*, sequences of six *fads2* promoters from five fish species were compared and analyzed (Figure S3), and the Sp1 binding site sequence in Δ6/Δ5 *fads2* promoter of *S.canaliculatus* was confirmed [23], then the corresponding sequence in Δ4 *fads2* promoter was mutated into the same sequence of Sp1 binding site in Δ6/Δ5 *fads2* promoter of *S. canaliculatus*. Dual luciferase assay showed that the Δ4 *fads2* promoter activity was significantly increased (Figure 8), which confirmed the importance of the Sp1 binding site for *fads2* promoter activity.

### 2.4. Knockdown of Sp1 Reduced Δ6/Δ5 fads2 and elovl5 mRNA Expression

**Figure 9:**
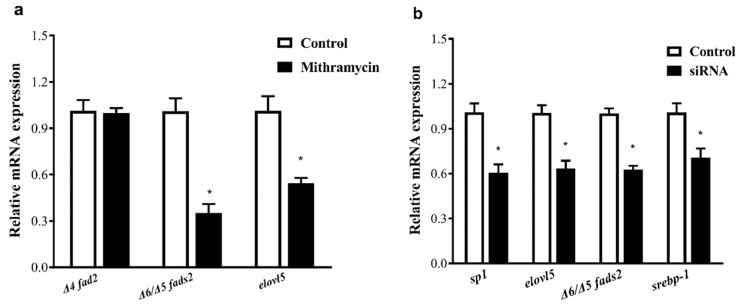
Q-PCR analyses of gene expression in *S. canaliculatus* hepatocyte line (SCHL) cells: (**a**) treated with the Sp1 antagonist mithramycin, (**b**) transfected with *sp1* siRNA or negative control siRNA (NC). Relative expression of the target genes in SCHL cells were quantified for each transcript and was normalized with the expression of 18S rRNA by 2^−ΔΔCt^ method. Results are means ± SEM (*n* = 3), * indicates significant differences compared with the control group using Student’s *t*-test at *p* < 0.05.

To determine the role of Sp1 in the regulation of Δ6/Δ5 *fads2* and *elovl5* expression, RNA interference and mithramycin A (a specific inhibitor of Sp1) were used to suppress *sp1* expression. When SCHL cells were exposed to 100 uM mithramycin A for 24 h, the mRNA levels of Δ6/Δ5 *fads2* and *elovl5* were significantly decreased, while that of Δ4 *fads2* showed no change (Figure 9a). Similarly, when *sp1*-siRNAs were used to transfect SCHL cells, depression of *sp1* mRNA expression was evident and, accordingly, the mRNA levels of Δ6/Δ5 *fads2, elovl5*, and *srebp-1* were significantly decreased (Figure 9b). These results further indicated that Sp1 up-regulated the expression of Δ6/Δ5 *fads2*, *elovl5*, and *srebp-1* mRNA.

### 2.5. Sp1 mRNA Overexpression Increased the Expression of Δ6/Δ5 fads2 and elovl5, and Enhanced LC-PUFA Biosynthesis in Rabbitfish SCHL Cells

**Figure 10:**
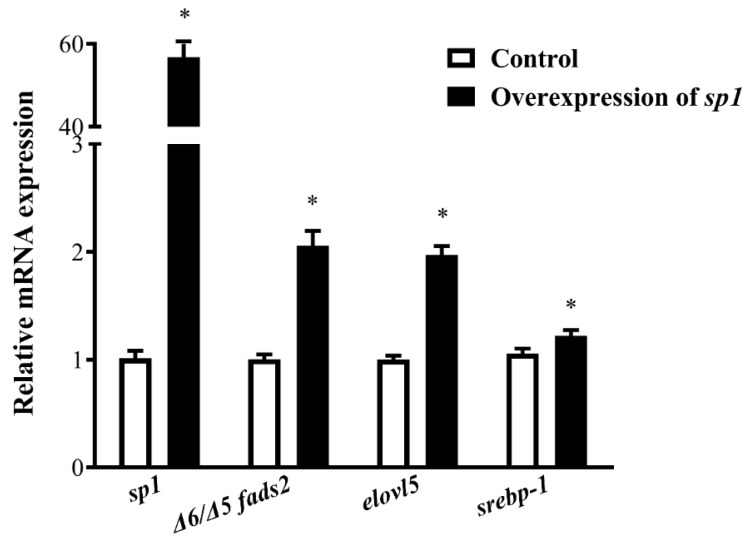
Q-PCR analyses of gene expression in SCHL cells transfected with *sp1* mRNA or control. Relative expression of the target genes was quantified for each transcript and was normalized with the expression of 18S rRNA by 2^−ΔΔCt^ method. Results are means ± SEM (*n* = 3), * indicates significant differences compared with the control group using Student’s *t*-test at *p* < 0.05.

The effect of Sp1 on the expression of Δ6/Δ5 *fads2* and *elovl5* was further confirmed in an overexpression experiment in SCHL cells. After rabbitfish *sp1* mRNA, which was synthesized in vitro, was transfected into SCHL cells, the mRNA of *sp1*, Δ6/Δ5 *fads2*, *elovl5*, and *srebp-1* were significantly increased (Figure 10), which suggested that overexpression of Sp1 increased the transcription of Δ6/Δ5 *fads2*, *elovl5*, and *serbp-1* genes.

Furthermore, the impact of Sp1 on LC-PUFA biosynthesis was determined by analyzing the fatty acid profiles of SCHL cells treated with *sp1* mRNA overexpression (Table 2). Fatty acid ratios of desaturation products/substrates such as 18:3n−6/18:2n−6 and that of elongation products/substrates such as 20:2n−6/18:2n−6 and 20:3n−3/18:3n−3, as well as the levels of DHA, EPA, ARA and total LC- were significantly increased, whereas the levels of C_18_ precursor 18:3n−3 (ALA) significantly decreased, with the *sp1* overexpression group compared with those in the control group. These results suggested that Sp1 improved the LC-PUFA biosynthetic ability of rabbitfish SCHL cells by enhancing the expression and enzymic activities of Fads2 and Elovl5.

## 3. Discussion

Sp1 is a transactivation molecule belonging to the family of Sp or Krüppel-like factor (KLF) proteins [26], and the Sp family of transcription factors is characterized by a particular combination of three conserved Cys2His2 zinc fingers [27]. In the present study, we cloned the rabbitfish *sp1* gene whose amino acid sequence shared high similarity and typical structural characteristics with those of other of other species. The C-terminus domain had the family marker region, featuring three Cys2His2 zinc fingers, required for sequence-specific DNA binding to GC-rich promoter elements [28,29]. Moreover, the sequences and structure were very similar between rabbitfish and zebrafish zinc finger domains, which suggested that rabbitfish Sp1 might also interact with GC sequences as found previously with zebrafish Sp1 [30]. Since the initial discovery of Sp1, it has generally been defined as a ‘basal’ transcription factor as single or multiple Sp1 binding sites have been mapped in promoters and enhancers of genes involved in almost all cellular processes. Besides, Sp1 plays an extremely important role in growth and metastasis of many tumors by regulating oncogenes, tumor suppressor genes, cell cycle control molecules, growth-related signal transduction, angiogenesis related factors, as well as apoptosis [28,31,32,33]. It is reported that suppression of *sp1* expression reduced the growth of colon cancer stem cells (CCSC) and induced apoptosis in vitro and in nude mouse xenografts, and the proportion of CCSC markers, CD44+/CD166+, was decreased following *sp1* knock-down [34]. Nevertheless, knowledge of the binding specificities of various Sp1 proteins for GC-boxes in promoter/enhancer DNA, or for other transcriptional and epigenetic regulators, is rather incomplete [35,36].

Several studies reported that Sp1 may be involved in the regulation of LC-PUFA biosynthesis in teleost fish by activating the promoter activities of genes encoding key enzymes although direct evidence was absent [22,23]. For instance, Sp1 elements were found in the *fads2* promoter regions of some fish species with LC-PUFA biosynthetic ability such as *S. salar* [22], *D. rerio*, and *S. canaliculatus* [23]. However, Sp1 elements were lacking in *fads2* promoter regions of carnivorous marine fish species like *G. morhua*, *D. labrax*, *L. japonicus*, *L. crocea* and *E. coioides*, in which LC-PUFA biosynthetic ability is lacking or very low. These data suggested that the lack of Sp1 binding sites may lead to low promoter activity of *fads2*, and thus result in low hepatic *fads2* expression in carnivorous marine teleosts, as recently shown in *E. coioides* [23]. Furthermore, it is reported that Sp3 is structurally similar to Sp1, with similar affinities for the Sp1-binding site [37]. Even so, their DNA-binding properties and regulatory functions are different [38]. There are several studies suggesting that Sp1 is responsible for basal transcription, and Sp3 is important for the induced transcription activation [39,40,41]. For example, the binding of Sp3 at the PKR promoter in vivo was interferon dependent, whereas the binding of Sp1 was constitutive [41]. Considering the complexity of the interaction between Sp1 and Sp3, the roles of Sp3 in LC-PUFA biosynthesis deserves further study.

While the above data suggested the importance of the Sp1-binding site in determining *fads2* promoter activity, the role of Sp1 in the transcriptional regulation of LC-PUFA biosynthesis in vertebrates was not directly shown. In the present study, potential Sp1 binding sites were found in rabbitfish *S. canaliculatus* Δ6/Δ5 *fads2* and *elovl5* promoters, but were not predicted in the Δ4 *fads2* promoter region. After the Sp1 binding site was inserted into the rabbitfish Δ4 *fads2* promoter, its activity was increased. Moreover, mutation of the Sp1 sites of Δ6/Δ5 *fads2* and *elovl5* promoters resulted in decreased promoter activities. These data provided direct evidence that Sp1 plays an important role in determining Δ6/Δ5 *fads2* and *elovl5* promoter activity in *S. canaliculatus*, and the weak activity of Δ4 *fads2* promoter may be, at least partly, due to the lack of Sp1 binding sites.

Sp1 can promote the expression of its target genes [42]. Generally, the level of gene transcription in eukaryotic cells is dependent on the binding of RNA polymerase and transcription factors to specific sequences in gene promoters [43]. Sp1 functions by interacting with the TATA-box binding protein complex (TFIID) and facilitating binding of TFIID to the promoter, which, in turn, recruits RNA polymerase II (Pol II) [29,44]. In addition, however, Sp1 also plays a key role in maintaining expression of genes that lack a TATA-box in the promoter [29,45]. As there was no TATA box in the promoters of rabbitfish *S. canaliculatus* Δ6/Δ5 *fads2* and *elovl5*, the significant changes in expression of Δ6/Δ5 *fads2* and *elovl5* when overexpressing or inhibiting *sp1* indicated that Sp1 can stimulate the expression of these two genes via the regulation of transcription activity. Further research is required to investigate the detailed regulation mechanisms of Sp1 on the expression of the rabbitfish *S. canaliculatus* Δ6/Δ5 *fads2* and *elovl5* gene.

It is reported that the fatty acid synthase (Fas) gene promoter is regulated by Sp1 and Srebp transcription factors [46]. Sp1 maintains the expression of *fas* directly and also has been shown to regulate Srebp-1c in colon cancer [47]. Srebp-1 is a member of the basic helix–loop–helix–leucine zipper family of transcription factors that regulate the biosynthesis of both cholesterol and fatty acids [48,49,50]. Previous studies indicated that Srebp-1 is a weak activator of transcription and only functions efficiently when activated by co-activating transcription factors such as SRE, E-box, LXR, NF-Y, and Sp1 [50,51,52]. Regulation of Srebp-1 by Sp1 has also been reported previously. For example, Sp1 functioned together with Srebp-1 to synergistically activate the *fas* promoter [53,54,55]. Similarly, studies of Srebps in fish including *S. salar* [22,56] and *D. labrax* [57] have reported previously that Srebp-1 mediated the expression of Δ6 *fads2*, and thus Srebp-1 may be involved in the transcriptional regulation of LC-PUFA biosynthesis in fish [22,58,59]. Interestingly, the highly conserved NF-Y and SRE elements were demonstrated in rabbitfish *S. canaliculatus* Δ6/Δ5 *fads2* promoter, suggesting Srebps as a major regulator of Δ6/Δ5 *fads2* expression [23,60]. The present study also indicated that Srebp-1 expression was changed when overexpressing or inhibiting Sp1. Therefore, Sp1 might also be indirectly involved in stimulating the expression of Srebp-1 to activate Δ6/Δ5 *fads2* and *elovl5* gene expression.

Sp1 enhanced LC-PUFA biosynthesis in SCHL cells by increasing Δ6/Δ5 *fads2* and *elovl*5 gene expression. Another study demonstrated that Sp1 binds to bovine *elovl7* promoter and activities the expression of *elovl7* in bovine mammary epithelial cells (bMECs) [21]. In rabbitfish, functional characterization showed that Δ6/Δ5 *fads2* could efficiently convert 18:2n−6 to 18:3n−6 [24] and the ratio of 18:3n−6/18:2n−6 is an index of Δ6 *fads2* activity [61]. In the present study, the expression of Δ6/Δ5 *fads2* and *elovl5* was increased by overexpression of *sp1* and, correspondingly, cell fatty acid profiles were changed. Overexpression of *sp1* was associated with increased levels of Δ6 desaturation products such as 18:3n−6 and elongation products such as 20:2n−6 and 20:3n−3, or with further downstream products in the LC-PUFA biosynthetic pathway such as ARA, EPA, and DHA. Moreover, overexpression of *sp1* increased the 18:3n−6/18:2n−6, 20:2n−6/18:2n−6, and 20:3n−3/18:3n−3 ratios in SCHL cells, which indicated that Sp1 could stimulate LC-PUFA synthesis in liver.

In summary, the present study demonstrated that Sp1 positively regulated the biosynthesis of LC-PUFA in rabbitfish, and functioned mainly through binding to Δ6/Δ5 *fads2* and *elovl5* promoters and activating their expression. To our knowledge, this is the first report of the direct involvement of Sp1 in the regulation of LC-PUFA biosynthesis at transcriptional and metabolic level in vertebrates, and this knowledge may contribute to efforts to enhance LC-PUFA biosynthesis in farmed fish.

## 4. Materials and Methods

### 4.1. Cell Cultures

The human hepatic carcinoma cell line (HepG2) was obtained from the China Center for Type Culture Collection (CCTCC, China) and cultured in DMEM (GlutaMAX) (Gibco, Life Technologies, Carlsbad, CA, USA) medium containing with 10% fetal bovine serum (FBS; Gibco, Life Technologies, Carlsbad, CA, USA) supplemented with 100 U/mL penicillin and 100 U/mL streptomycin at 37 °C with 5% CO_2_ [62]. The rabbitfish *S. canaliculatus* hepatocyte cell line (SCHL) was grown at 28 °C using DMEM-F12 (Gibco, Life Technologies, Carlsbad, CA, USA) medium supplemented with 10% FBS and 0.5% rainbow trout *O. mykiss* serum (Caisson Labs; www.caissonlabs.com) [63].

### 4.2. RNA Isolation and cDNA Synthesis

Total RNA was extracted from the liver of rabbitfish *S. canaliculatus* with Trizol reagent (Invitrogen, Carlsbad, CA, USA). The concentration of RNA samples was measured using a NanoDrop 2000 (Thermo Scientific, Carlsbad, CA, USA) and quality confirmed by agarose gel electrophoresis (Figure S2.). The cDNA was synthesized from the template of 1 μg RNA using High-Capacity cDNA Reverse Transcription Kits (Thermo Scientific, Carlsbad, CA, USA) for partial sequence cloning of *sp1* or gene expression analysis.

### 4.3. Cloning of the Full-Length sp1 cDNA in Rabbitfish

Primers *sp1*-ZLS and *sp1*-ZLA were designed according to the transcriptome data of rabbitfish and used for amplifying partial sequences of the putative *sp1* cDNA (Table 3). The PCR program was set as follows: initial denaturation 94 °C for 3 min, 35 cycles of denaturation at 94 °C for 30 s, annealing at 55 °C for 30 s and extension at 72 °C for 2 min, followed by a final extension at 72 °C for 5 min. Specific primers, *sp1*-3RACE outer/*sp1*-3RACE inter and *sp1*-5RACE outer/*sp1*-5RACE inter, were designed to produce the full-length *sp1* cDNA through 5′ and 3′ rapid amplification of cDNA ends (RACE) PCR (SMART-RACE cDNA Amplification Kit, Takara, Tokyo, Japan) (Table 3) and PCR was performed according to the manufacturer’s instructions (Takara, Tokyo, Japan). The annealing temperature was 62 °C for extending sequences. The PCR products were purified by gel recovery and inserted into the pEASY-Blunt Cloning Kit (TRANS Gene, Beijing, China) for further sequencing (Sangon Biotechnology Company, Shanghai, China).

### 4.4. Phylogenetic Analysis of Cloned Sp1 Sequence

The *sp1* sequence was analyzed with software DNAman 6.0, and putative amino acid sequences predicted by ORF Finder (http://www.ncbi.nlm.nih.gov/gorf/gorf.html). Amino acid sequences of other vertebrate Sp1 were obtained from protein databases (NCBI) for alignments and constructing phylogenetic trees and the identities of sequences blasted by Blastp (http://blast.ncbi.nlm.nih.gov/). The neighbor joining (NJ) method (bootstrap method: 1000 replications) was used to perform multiple alignments using MEGA 5.0 software. The secondary and three-dimensional (3D) structures of Sp1 were predicted by PredictProtein (http://www.predictprotein.org/) and SWISS-MODEL (http://swissmodel.expasy.org/) [64], respectively.

### 4.5. Bioinformatic Analysis

The rabbitfish Δ6/Δ5 *fads2* and *elovl5* promoters were cloned from genomic DNA of *S. canaliculatus*, and the corresponding core promoter regions were respectively located at −456 to +51 bp and −837 to +89 bp as reported previously [65,66]. TFBIND^®^, TRANSFAC^®^, PROMO^®^, and JASPAR^®^ databases were used to predict the binding elements of transcription factors in the promoters. The Sp1 binding element mutations were conducted in the promoter regions of −159 to −137 in Δ6/Δ5 *fads2* and −491 to −468 in *elovl5*, respectively. The promoter structure was highly conserved between Δ4 *fads2,* Δ6/Δ5 *fads2*, and the Sp1-binding sites were predicted in promoter region of Δ6/Δ5 *fads2* but not in that of Δ4 *fads2* in *S. canaliculatus*.

### 4.6. Effects of Candidate Sp1 Elements on Rabbitfish Δ6/Δ5 fads2, Δ4 fads2 and elovl5 Promoters

To determine the potential effect of the predicted Sp1 binding sites on promoter activity, the promoter reporter vector was constructed with the Δ6/Δ5 *fads2* and *elovl5* promoter fragment and pGL4.10, and the Sp1 binding sites-directed mutant of the Δ6/Δ5 *fads2* and *elovl5* promoter were constructed with the mutation site in the middle of the primer. Mutations of *S. canaliculatus* Δ6/Δ5 *fads2* and *elovl5* promoter were performed with Muta-direct^TM^ site-directed mutagenesis kit (SBS Genetech, Shanghai, China) according to the manufacturer’s protocol. Constructs D2 and D3 including the Δ6/Δ5 *fads2* and *elovl5* core promoter region were used as wildtype for mutations experiments and the site-directed mutation plasmids from D2 and D3 were designated D2M and D3M, respectively. In order to clarify the regulatory mechanisms of Sp1 on Δ4 *fads2*, the Sp1 binding site sequence in Δ6/Δ5 *fads2* promoter of *S. canaliculatus* was confirmed and then inserted into the corresponding location of the Δ6/Δ5 *fads2* promoter [23]. The detailed strategy of site-directed mutation and the primers of targeted mutation are shown in Table 1 and Table 4. All plasmid constructs were confirmed by sequencing (Sangon Biotechnology Company, Shanghai, China).

HepG2 cells were seeded in 96-well plates (Eppendorf, Hamburg, Germany) 24 h before transfection, then transfected with 100 ng of each reporter firefly luciferase construct, and co-transfected with 0.05 ng of vector pGL4.75 (Promega, Madison, WI, USA) and Lipofectamine^®^ 2000 Reagent (0.25 μL) (Invitrogen, Carlsbad, CA, USA) according to the manufacturer’s instructions. The empty vector pGL4.10 with no promoter sequence was treated as a negative control in each transfection assay. The promoter reporter vector contained the Firefly luciferase gene. Luciferase assays were performed 48 h after transfection with the Dual-Glo^TM^ luciferase assay system (Promega, Madison, WI, USA), and chemical luminescence intensity detected in duplicate readings using a microplate reader (Infinite M200 Pro, Tecan, Switzerland). Promoter activity was calculated from the chemical luminescence intensity ratio of firefly: Renilla luciferase for each construct, and then compared with the activity of vector pGL4.10 luciferase.

### 4.7. Electrophoretic Mobility Shift Assay (EMSA)

To confirm the binding of Sp1 to the promoters of rabbitfish Δ6/Δ5 *fads2* and *elovl5*, nuclear protein was extracted from SCHL cells with Nucleoprotein Extraction Kit (Beyotime, Shanghai, China). The cell lysates were ultracentrifuged at 12,000× *g* for 10 min at 4 °C. The clear supernatants were collected as the cytoplasmic fraction. For precipitation, the residual supernatant was completely absorbed and 50 μL of nucleoprotein extraction reagent with PMSF was added. Then centrifuging for another 10 min. The clear supernatants were collected as the nucleoprotein extracted. The 26 and 32 bp 5′ end biotin-labeled probes covering the predicted Sp1 elements were designed and incubated with the proteins to determine whether Sp1 interacted with the promoters of Δ6/Δ5 *fads2* and *elovl5*. Both the labeled and unlabeled probes were obtained from Shanghai Sangon Biotech. The effects of biotinylated DNA binding to SCHL cells nuclear protein was detected by EMSA using LightShift™ chemiluminescent EMSA kit (Beyotime, Shanghai, China) according to the manufacturer’s instructions. Briefly, the reaction system consisted of 20 fmol of biotin-labeled oligonucleotides and the control group was supplemented with 200-fold excess of competitor/competitor-mutation oligonucleotides. After incubation, the mixtures were run on polyacrylamide gels and transferred onto nylon membrane and analyzed with Odyssey Fc imaging system (Li-Cor, Nebraska, USA). Detailed information of the oligonucleotide probes is shown in Table 5.

### 4.8. Mithramycin A Treatment to SCHL Cells

Mithramycin A (Sigma-Aldrich, St. Louis, MO, USA) is a specific inhibitor of Sp1, which could block Sp1-mediated transcription by preventing its binding to the GC rich region in the promoter [67]. The SCHL cells were seeded in 6-well plates (Eppendorf, Hamburg, Germany) and incubated for 24 h to 80% confluence, and then the cell culture medium was replaced with DMEM with 10% FBS containing 100 nM mithramycin A (final concentration) for 24 h [68]; the control group was treated with the same volume ddH_2_O. Each treatment was conducted in triplicate wells as technical replicates. The cells were harvested using Trizol reagent, followed by RNA isolation according to the manufacturer’s instruction (Invitrogen, Carlsbad, CA, USA) as described above.

### 4.9. Effect of siRNA on sp1, Δ6/Δ5 fads2 and elovl5 Gene Expression in SCHL Cells

To further clarify the influence of Sp1 on Δ6/Δ5 *fads2* and *elovl5* regulation, small interference RNA fragments (siRNA) targeting the rabbitfish *sp1* was run by transfection into SCHL cells. siRNA of *sp1* were synthesized (GenePharma, Shanghai, China) by using the primer pairs (Table 6). The SCHL cells were seeded in 6-well plates for 24 h, then transfected with 100 pmol/well siRNA by Lipofectamine^®^ 2000 Reagent (Invitrogen, Carlsbad, CA, USA). siRNA was set as the experiment group and negative control (NC) as the negative control (Table 6). The cells were harvested 48 h after transfection for quantitative real-time PCR (qPCR) analyses.

### 4.10. Influence of sp1 mRNA Overexpression on sp1, Δ6/Δ5 fads2 and elovl5 Gene Expression in SCHL Cells

The influence of Sp1 on Δ6/Δ5 *fads2* and *elovl5* expression was further established by running an mRNA overexpression assay performed by transfecting *sp1* mature transcripts into SCHL cells. mRNA transcription in vitro was performed on a linearized DNA template containing T7 promoter and rabbitfish *sp1* cDNA sequence using mMESSAGE mMACHINE T7 Ultra Kit (Ambion, Carlsbad, CA, USA). Overexpression vector pcDNA3.1(+)-*sp1* was used to synthesis a linearized DNA template with sense primer (T7 promoter) and antisense primer (*sp1* reverse primer) in *Pfu*-PCR reaction (Table 7). Finally, the *sp1* mRNA product was purified with MEGAclear^TM^ Kit (Ambion, Austin, TX, USA), and stored in −80 °C for further transfection into SCHL cells.

The SCHL cells were seeded in 6-well plates for 24 h, then transfected with 250 ng *sp1* mRNA by Lipofectamine^TM^ Messenger MAX^TM^ Reagent (Ambion, Carlsbad, CA, USA) per well. After 24 h incubation, the cells were collected and lysed for RNA isolation prior to qPCR analysis. After 48 h incubation, cells were collected and lysed for lipid extraction prior to analysis of fatty acids composition.

### 4.11. Lipid Extraction and Fatty Acid Analysis by Gas Chromatography Spectrometer

The impact of Sp1 on LC-PUFA biosynthesis was determined by analyzing the fatty acid profiles of SCHL cells treated with *sp1* mRNA overexpression. The cells were treated with trypsin-EDTA (Invitrogen, Carlsbad, CA, USA), centrifuged at 4000× *g* for 5 min, and cell pellets collected for lipid extract using chloroform/methanol (2:1, *v*/*v*). Fatty acid methyl esters (FAME) were prepared by transesterification with boron trifluoride etherate (ca. 48%, Acros Organics, NJ, USA) [69] and separated using a gas chromatograph spectrometer (GC2010-plus, Shimadzu, Japan) as described in detail previously [70]. Samples were analyzed in triplicate.

### 4.12. Quantitative Real-Time PCR Analysis

Tissue distribution of *sp1* mRNA and the expression of *sp1*, Δ6/Δ5 *fads2*, Δ4 *fads2*, *elovl5*, and *srebp-1* from experiments involving SCHL cells were analyzed by qPCR analysis, and primer information is shown in Table 8. Total RNA was isolated and reverse transcribed to obtain cDNA as described above. Each qPCR (total volume of 20 μL) consisted of 2 μL diluted cDNA (10 ng/μL), 0.5 μM of each primer, and 10 μL SYBR Green I Master (Invitrogen, Carlsbad, CA, USA). The qPCR procedures consisted of an activation step at 94 °C for 5 min and 40 cycles at 95 °C for 10 s, 61 °C for 30 s, and 72 °C for 20 s; subsequently, melting curves were plotted to confirm amplification of a single product in each reaction. The relative RNA levels of genes in each sample were normalized with 18S rRNA (GenBank: AB276993) expression calculated by the comparative threshold cycle (Ct) method [71]. The qPCR reactions were carried out on the Lightcycler 480 system (Roche, Basel, Switzerland). Triplicate wells were used per sample and three independent experiments performed.

### 4.13. Statistical Analysis

Data were presented as means ± SEM. Differences among groups were analyzed by one-way analysis of variance (ANOVA) followed by Tukey’s multiple comparison test or Student’s *t*-test (as indicated) at a significance level of *p* < 0.05 using OriginPro 7.5 software (OriginLab Corporation, Northampton, MA, USA).

## Figures and Tables

**Table 1:** Sp1 binding sites predicted in the promoter of rabbitfish *S. canaliculatus* Δ6/Δ5 *fads2* and *elovl5* using online software and site-directed mutation sites.

Gene	Position	Predicted Element	Mutation Site
Δ6/Δ5 *fads2*	−159 ~ −137	TGGGCGCGC	GG→TT
*elovl5*	−491 ~ −468	TGTCCCTCCTTTT	CC→AA
Δ*4 fads2*	−187 ~ −164	TGGCAACTG	CAACTG→GCGCGC

The position of each element is numbered relative to the presumed the transcription start site (TSS). The bases underlined are the mutation sites for site-directed mutant.

**Table 2:** Fatty acids composition of the rabbitfish *S. canaliculatus* hepatocyte line cells treated with *sp1* mRNA overexpression or control ^1^.

Main Fatty Acids (% Total Fatty Acid)	Control	Overexpression *sp1*
14:0	1.41 ± 0.09	1.31 ± 0.17
14:1	0.32 ± 0.02	0.40 ± 0.09
16:0	24.61 ± 0.20	25.49 ± 4.61
16:1	0.30 ± 0.02	0.23 ± 0.03
18:0	19.91 ± 0.90	20.34 ± 3.90
18:1	25.95 ± 1.58	22.86 ± 1.40
18:2n−6 (LA)	4.08 ± 0.20	3.42 ± 0.30
18:3n−6	0.07 ± 0.01	0.13 ± 0.01
20:1	0.43 ± 0.03	0.60 ± 0.12
18:3n−3 (ALA)	0.46 ± 0.05	0.20 ± 0.03 *
20:2n−6	0.24 ± 0.02	0.24 ± 0.01
22:0	0.24 ± 0.02	0.19 ± 0.05
20:3n−6	1.84 ± 0.15	1.98 ± 0.15
22:1n−9	0.49 ± 0.01	0.40 ± 0.06
20:3n−3	0.15 ± 0.01	0.15 ± 0.01
20:4n−6 (ARA)	6.10 ± 0.38	7.52 ± 0.26 *
22:2n−6	0.51 ± 0.01	0.47 ± 0.01
20:5n−3 (EPA)	2.11 ± 0.21	2.77 ± 0.04 *
24:1n−9	0.21 ± 0.01	0.19 ± 0.02
22:6n−3 (DHA)	9.90 ± 0.40	11.50 ± 0.31 *
ΣLC-PUFA	12.15 ± 0.70	14.84 ± 0.36 *
18:3n−6/18:2n−6	0.09 ± 0.002	0.13 ± 0.01 *
20:2n−6/18:2n−6	0.06 ± 0.001	0.09 ± 0.01 *
20:3n−3/18:3n−3	0.20 ± 0.01	0.74 ± 0.12 *

^1^ Results are means ± SEM (*n* = 3). Values in each row with * indicate significant difference (analyzed by ANOVA followed by paired *t*-test; *p* < 0.05).

**Table 3:** Primers for cloning *sp1* in this study.

Primers	Nucleotide Sequence
*sp1*-ZLS	GGATGTACTGGGAGGATCTGTA
*sp1*-ZLA	GAAGAAGACCTCGGTGGATATTG
*sp1*-5RACEouter	AAGTTTGCTTGCCCAGAGTGTCC
*sp1*-3RACEinter	ACCTTCACCAAATAAACGGCAACAACT
*sp1*-5RACEouter	CGGATGGCGAAAGCACCTGTCTG
*sp1*-5RACEinter	GATGCTTGAACACTGAGAGGAATAACC

**Table 4:** Primers used for site-directed mutations of Sp1 binding sites.

Targeted Mutation	Primers	Nucleotide Sequence
Δ6/Δ5 *fads2*	6pmSp1-S	TGTTTCCGTCTGGCAACGCAGGCGACGTTT
	6pmSp1-A	AAACGTCGCCTGCGTTGCCAGACGGAAACA
*elovl5*	5pmSp1-S	TCAAACATTCCCTTGTCAATCCTTTTTTGAGCTA
	5pmSp1-A	TAGCTCAAAAAAGGATTGACAAGGGAATGTTTGA
Δ4 *fads2*	4pmSp1-S	CATCGGACTTGGGCGCGCCCTCCTTATTAT

Details of binding sites for TFs are shown in Table 1. The bases underlined are chosen for site-directed mutant.

**Table 5:** Probes used for electrophoretic mobility shift assay (EMSA).

Aim	Primers	Nucleotide Sequence
**Δ6/Δ5 *fads2* EMSA probes**	6B-S (5′-biotin labeled)	GTTTCCGTCTGGGCGCGCAGGCGACG
	6B-A (5′-biotin labeled)	CGTCGCCTGCGCGCCCAGACGGAAAC
	6U-S (5′ unlabeled)	GTTTCCGTCTGGGCGCGCAGGCGACG
	6U-A (5′ unlabeled)	CGTCGCCTGCGCGCCCAGACGGAAAC
***elovl5* EMSA probes**	5B-S (5′-biotin labeled)	TCAAACATTCCCTTGTCCCTCCTTTTTTGAGCTA
	5B-A (5′-biotin labeled)	TAGCTCAAAAAAGGAGGGACAAGGGAATGTTTGA
	5U-S (5′ unlabeled)	TCAAACATTCCCTTGTCCCTCCTTTTTTGAGCTA
	5U-A (5′ unlabeled)	TAGCTCAAAAAAGGAGGGACAAGGGAATGTTTGA

**Table 6:** RNAi nucleotide sequence used in this study.

RNA Interference	Primers	Nucleotide Sequence
Negative control	NC-S	UUCUUCGAACGUGUCACGUTT
	NC-A	ACGUGACACGUUCGGAGAATT
*sp1* interference	siRNA-S	CCGGGCAUUUCAGAGUAATT
	siRNA-A	UUACUCUGAAAUGUCCCGGTT

**Table 7:** Primers for *sp1* overexpression.

Primers	Nucleotide Sequence
T7 promoter primer	TAATACGACTCACTATAGGG
*sp1* reverse primer	TTAGAAGTTGTTGCCGTTTATTTGGT

**Table 8:** Primers used for qPCR.

Aim	Primers	Nucleotide Sequence
*sp1*	QS-*sp1*	CCACTTCCTCCTCTTATGGAATG
	QA-*sp1*	ATCTCTGTTGTCTGGCTGTATG
Δ6/Δ5 *fads2*	QS-D6 *fads*	AACACCATTTGTTTCCCACC
	QA-D6 *fads*	CAGTGACCTGATGATATCAGCG
Δ4 *fads2*	QS-D4 *fads*	GAACACCATTTGTTCCCGAG
	QA-D4 *fads*	TTCAGTGCCCTGACGACG
*elovl5*	QS-*elovl5*	GCACTCACCGTTGTGTATCT
	QA-*elovl5*	GCAGAGCCAAGCTCATAGAA
*srebp-1*	QS-*srebp-1*	AGCCAGACACAAGAGGAAAG
	QA-*srebp-1*	AAGAGGGCCGTGTCAATATC
18s RNA	QS-18SrRNA	CGCCGAGAAGACGATCAAAC
	QA-18SrRNA	TGATCCTTCCGCAGGTTCAC

## Supplementary Materials

Supplementary materials can be found at https://www.mdpi.com/1422-0067/20/20/5066/s1.

## Abbreviations

ALA	α-linolenic acid (18:3n−3)
ARA	arachidonic acid (20:4n−6)
DHA	docosahexaenoic acid (22:6n−3)
EFA	essential fatty acid
Elovl	elongase of very long-chain fatty acids
EMSA	electrophoresis mobility shift assay
EPA	eicosapentaenoic acid (20:5n−3)
FAS	fatty acid synthase
HepG2	liver hepatocellular cell line
Ppar	peroxisome proliferator-activated receptor
PUFA	polyunsaturated fatty acid
SCHL	*Siganus canaliculatus* hepatocyte cell line
siRNA	small interfering RNA
Sp1	stimulatory protein 1
Srebp	sterol regulatory element binding protein
TF	transcription factor
TSS	transcription start site
