# Supplementary material for: Sp1 is Involved in Vertebrate LC-PUFA Biosynthesis by Upregulating the Expression of Liver Desaturase and Elongase Genes
Source: Int J Mol Sci. 2019 Oct 12;20(20):5066. doi: 10.3390/ijms20205066 (PMC6829471; doi:10.3390/ijms20205066)
Supplement: Supplementary file 1 [file ijms-20-05066-s001.pdf]

Supplemental Figures

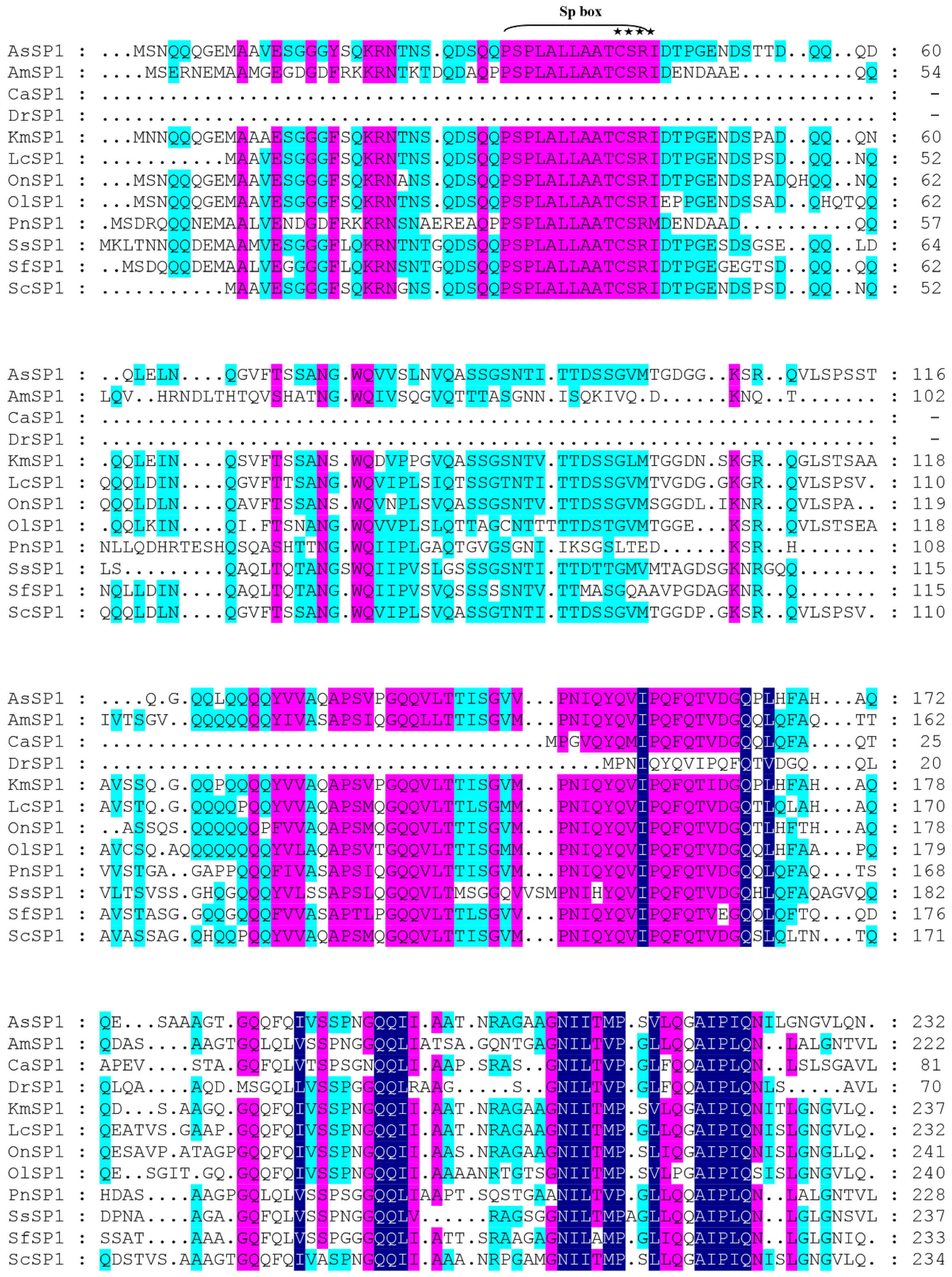

```
AsSP1 : ..QPQFL.ANMPVSLNGNITLV.PVSTGVTGGDANS...GETS.GNQLIQQSP..QPVS....SNNGT : 286
AmSP1 : P.SQTQF.LANVPMSLNGGITLLPVSAGQAAGAKADDGTS..GVG...Q..QVV..QQA....IVS.. : 273
CaSP1 : P.NQAQF.LTNMPL..NANITLLPVGSV..................SDA..NAGGAPQQ....LLQ.. : 119
DrSP1 : P.NQTQF.LANVPL..NANITLLPVGPGPVG.....................GDTHTA....VAA.. : 106
KmSP1 : ..NQPQF.LANMPVSLNGNITLLPVSTGATGGDANGN..GDTG.GNQLIQQQSKQPVS....SNSGA : 295
LcSP1 : ..NQPQF.LANMPVSLNGNITLLPVSTGASGTGDANS.GGDTG.GNQLI..QQSQHPVS....SNS.. : 287
OnSP1 : ..NQPQF.LANMPMSLNGNITLLPVTAGAA..GGDTNGGGEAG.GNQLM..QQQQQP.VSSNSEA.. : 298
OlSP1 : ..NQPQI.LANMPVSLNGNITLLPVSTGATGGDANCA..GDGG.GHQQVEN.QTQQPVS......SGA : 295
PnSP1 : N.NQTQF.LANMPVSLNGSITLLPVSAGAAAGAKSDDVTS..GTSGVSQ..QLLHAQQA....VAA.. : 284
SsSP1 : QNNQGQFQLANMPGLLNGNITLLPVSAASGSEGDGGSN..........Q..QLMQQVVS....TS... : 286
SfSP1 : ..NQAQF.LANMPVSINGNITLLPVATGTTGGGGDAAGV..G.SGSSQ..QLLQQPAS....SAG.. : 287
ScSP1 : ..NQPQF.LANMPVSLNGNITLLPVSTGTTGTGADANGGGDAG.GNQLV..QQQQLQNAVSSNSNT.. : 294

AsSP1 : SYMTSESTITT.QTT...SSYGI.TTQKSNGSGS.GTFQQNPASSLGIPIQPDNRAE.......QQPQ : 341
AmSP1 : TATEYHTSATTSTTQAVMSCSGGVSRANTVTGNTFQTTVGTIP...IQAIQSDNRDGKQQQQQQQQQT : 338
CaSP1 : STTAAEYCTTSTSTQTTAPGGVLTLAQSNTSEPGKTFQNTSGD...GPKVSQAQIVI.........QP : 175
DrSP1 : PPQGPEFCSTASSTRATAAVGIATLAQSRA................PAASDGQKG..........QP : 147
KmSP1 : GYMTSASTVTT.QAT...SSYGI.TQTQKSNGSVTGTFQQNPTTSLGVPIQPDTRDG.......QQPQ : 351
LcSP1 : GYMTSASTITT.QAS...TSYGM.TQTQNTNGVMTGTFQHNAATSLGVPIQPDNRDG.......QQPQ : 343
OnSP1 : GYMTSASTVTTQT.S...SSYGV.TQKQNSNGAVTGTFQQNMASSLGVPIQPDNRDR.......GQPQ : 354
OlSP1 : GYMTNVSTVTT.Q.T...TSYGI.TQTQKNNGSGTGSFQQSPS.SVGVAVQPENRNG.......QQPQ : 349
PnSP1 : PVTAEYHSSSTTSTTQVMTSCSGGVSSQSNTM.TGATFQTTAA...GISVQSENRDGK........QP : 340
SsSP1 : ..GTYYTNSTTTTTQTGTSYGGMTQQTQSTNGFQNSGGGIPIQ...PDNRD................ : 332
SfSP1 : GTGFYTNTITTTTTSQAATSYGGGTQAQSSSGVGGNGFQSSGG...GASGQSDNRDG........QN : 343
ScSP1 : GYMTSASTVTT.T.S...SSYGM.TQTQNSNVAMTGTFQGNTGSSLSVPIQPDNRDG.......QQPQ : 349

AsSP1 : QIL.........IQSPQVIQGGAPLQTIQASTVTSAGGQVFAAPTLSQEGLQNLQIM..PNTGPILLR : 398
AmSP1 : QQQPQI.....LIQPQQLFQGTSTLQAIQPA.....SGQVFATQTLSQDGLQNLQM..IPNNSSILLR : 394
CaSP1 : QQVLQS.....VSA.....................GGQVFATQSLSQDGLQNVQIQTIANGSPILIR : 216
DrSP1 : QMLLQGVQQGAVSA.....................PGPVFTTQTLSQDGVQNVQIQTIASGSPILIR : 193
KmSP1 : QIL.........IQPQQVIQGGTSLQTIQASAVATAGGQVFATPTLSQEGLQNLQIM..PNTGPILLR : 408
LcSP1 : QIL.........IQPQQVIQGGTPLQTIQAGTVATAGGQVFAAPTLSQEGLQNLQIM..PNAGPILLR : 400
OnSP1 : QIL.........IQPQQVIQGGAQLQTIQAGTVAATGGQVFTPTLSQEGLQNLQIMP..NTGA.ILLR : 410
OlSP1 : QIL.........IQPQQVIQGGTSLQTIQ......AGGQVFAAPTLSQEGLQNLQIM..PNTGPILLR : 400
PnSP1 : QQHPQV.....LIQPQQIFQGAPALQAIQSG.....GGQVFAAHTLSQEGLQNLQIQTIPNNSTILLR : 398
SsSP1 : .QLQQI.....LIQPQQLIQGGTSLQTISA......GGQVFATPTLTQDALQNLQIQTIPNTSPILLR : 388
SfSP1 : QQPQQI.....VIQPQQLLQSGHSLQTIQTGAIST.GGQVIAAQTLSQDALQNLQIQAIPNTSPILVR : 405
ScSP1 : QIL.........IQPQQVIQGGAPL...QAGTVATAGGQVFATPTLSQEGLQNLQIM..PNTGPILLR : 403

                 ★★★★★
AsSP1 : TVGPNGQVSWQTIHIQNPAGAQITLAPVQSLPQLG....QTQGG..TGAGTVP..V.QIPNLQTINLN : 457
AmSP1 : TVGPNGQVSWQTVQLQSPAGAQITLAPVHSLPQLTQTGTTAT.TMQ...............LPGLQTI : 446
CaSP1 : TVGPNGQVSWQTLQLQSPANTQITLAQ...........................PGTLSGLQTINLN : 256
DrSP1 : TLGADGQVSWQTLQLQSPGA.QITLAPTGASG......................PVQLSGLQTINLN : 237
KmSP1 : TVGPNGQVSWQTIQIQSPAGTQITLAPVQSLPQLG....QPQGG..AGAVPVS..TMQIPSLQTINLN : 468
LcSP1 : TVAPNGQVSWQTIQIQSPAGPQITLAPMQSLPQLG....QAQGAAAAGGVSVN..TVQIPGIQTINLN : 462
OnSP1 : TVAPNGQVTWQTIQIQNPTGAQITLAPVQSLPQLG....QTQGTAAAGGVPVN..TVQIPGIQTINLN : 472
OlSP1 : TVGPNGQVSWQTIQIQSPTGAQIALAPVQSISQLG....QAQGN..TGAGAVPVNTLQIPGIQTINLN : 462
PnSP1 : TVGPNGQVSWQPVQLQSPAGAQITLAPVPSLPQLAQTG.TAT.TMQ...............LPGLQTI : 449
SsSP1 : TVGLNGQVSWQTLQLQSPAGTQITLAPM...QGLSQLG.QAQ.GGTM..........QLPGLHTINLN : 441
SfSP1 : TLGPNGQVSWQTLQLQSPAGAQITLAPVQSLQPLPQLA.QAQ.AGSAGGVSVN..PVQLPGLQTINLN : 469
ScSP1 : TVGPNGQVSWQTIQIQSPTGPQITLAPVQSLPQLG....QAQGAAAPGGVSVN..TVQIPGIQTINLN : 465
```

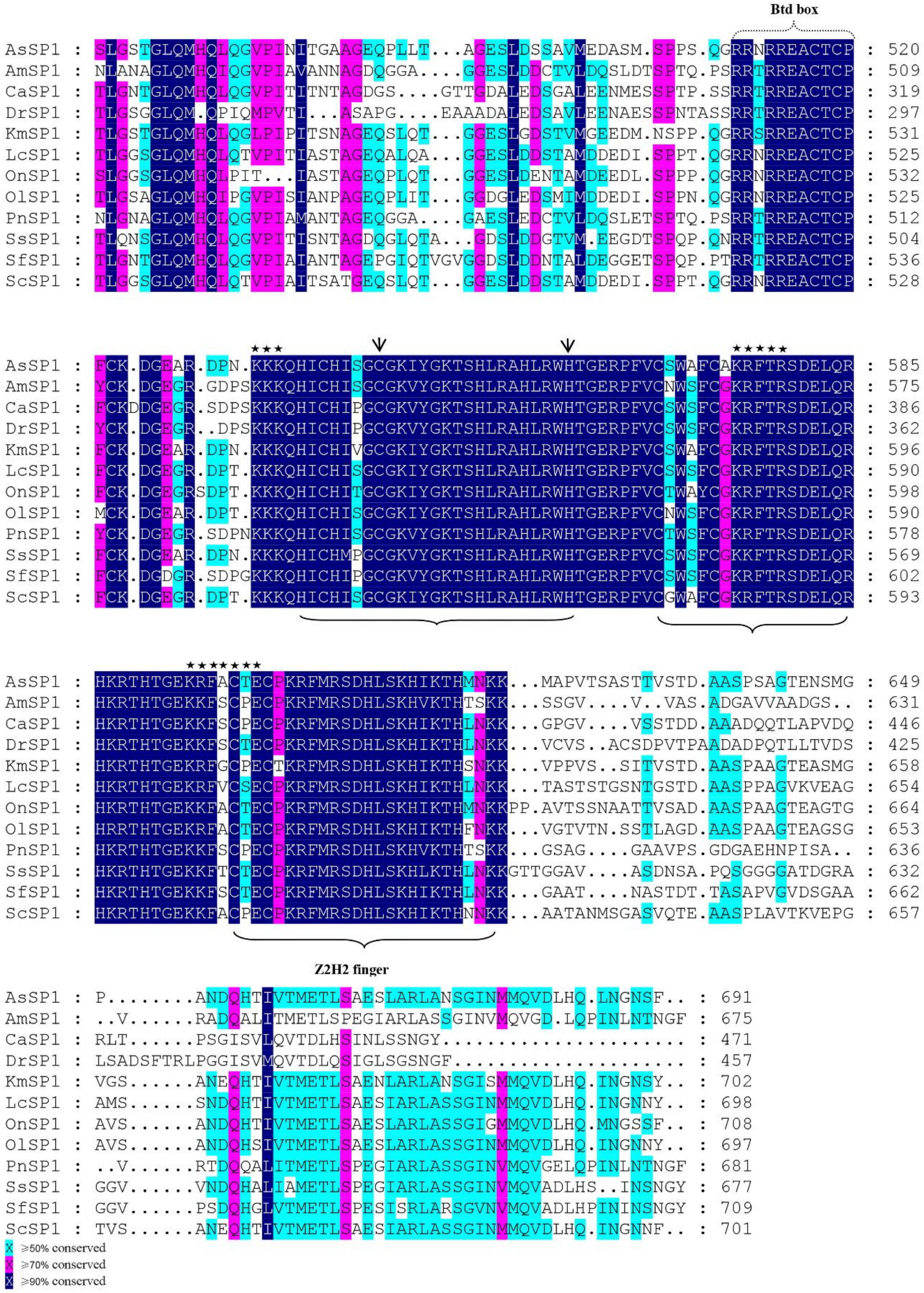

Figure S1. Alignments of the deduced full-length amino acid (aa) sequences of Sp1 from *S. canaliculatus* (ScSP1) and other fish species (*L. crocea*, LcSP1, XP_010730401.1; *S. salar*, SsSP1, XP_013989519.1; *A. striatum*, AsSP1, SBP16265.1; *A. mexicanus*, AmSP1, XP_007248419.1; *K. marmoratus*, KmSP1, XP_017264015.1; *O. niloticus*, OnSP1, XP_019214905.1; *O. latipes*, OlSP1, XP_004068725.1; *P. nattereri*, PnSP1, XP_017548304.1; *S. formosus*, SfSP1, XP_018597836.1; *D. rerio*, DrSP1, AAH67713.1). The black and gray boxes indicate identical and similar aa residues, respectively. The solid-line and dotted-line brackets indicate Sp and Btd boxes, respectively. The solid

braces denote zinc finger domains. The pentagrams and arrows indicate potential phosphorylation sites and Zn binding sites.

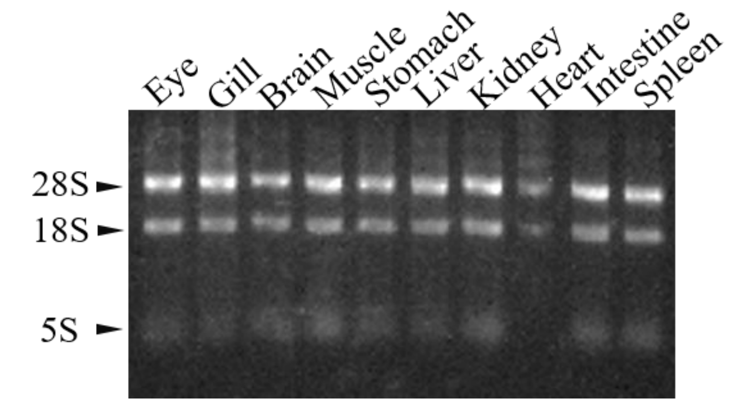

Figure S2. RNA integrity assay for eye, gill, brain, muscle, stomach, liver, kidney, heart, intestine and spleen of rabbitfish (*S. canaliculatus*), related to Figure 3.

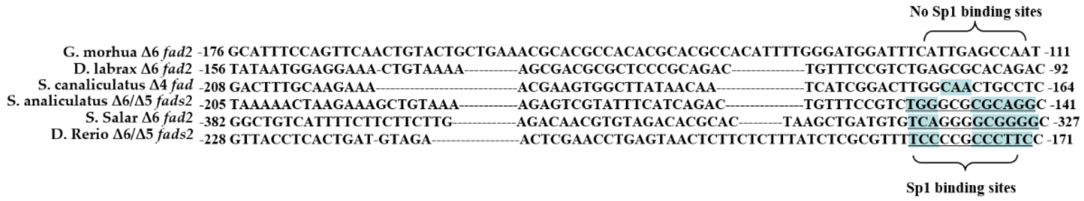

Figure S3. Alignment of *fads2* promoters among rabbitfish (*S. canaliculatus*) and other fish species. The numbers indicate sequence positions relative to possible transcription start site, related to Figure 8.
